# Thermosensitive Hydrogel for Encapsulation and Controlled Release of Biocontrol Agents to Prevent Peanut Aflatoxin Contamination

**DOI:** 10.3390/polym12030547

**Published:** 2020-03-03

**Authors:** Jiachang Feng, Jianpeng Dou, Youzhen Zhang, Zidan Wu, Dongxue Yin, Wenfu Wu

**Affiliations:** Department of Biological and Agricultural Engineering, Jilin University, Changchun 130000, China; fengjc17@mails.jlu.edu.cn (J.F.); doujp@jlu.edu.cn (J.D.); youzhen18@mails.jlu.edu.cn (Y.Z.); wuzidan@jlu.edu.cn (Z.W.); yindx17@mails.jlu.edu.cn (D.Y.)

**Keywords:** thermosensitive, biocontrol, aflatoxin, PNIPAAm, encapsulation

## Abstract

Starch, alginate, and poly(*N*-isopropylacrylamide) (PNIPAAm) were combined to prepare a semi-interpenetrating network (IPN) hydrogel with temperature sensitivity. Calcium chloride was used as cross-linking agent, the non-toxigenic *Aspergillus flavus* spores were successfully encapsulated as biocontrol agents by the method of ionic gelation. Characterization of the hydrogel was performed by Fourier-transform infrared spectroscopy (FTIR), scanning electron micrograph (SEM), and thermogravimetry analysis (TGA). Formulation characteristics, such as entrapment efficiency, beads size, swelling behavior, and rheological properties were evaluated. The optical and rheological measurements indicated that the lower critical solution temperature (LCST) of the samples was about 29–30 °C. TGA results demonstrated that the addition of kaolin could improve the thermal stability of the semi-IPN hydrogel. Morphological analysis showed a porous honeycomb structure on the surface of the beads. According to the release properties of the beads, the semi-IPN hydrogel beads containing kaolin not only have the effect of slow release before peanut flowering, but they also can rapidly release biocontrol agents after flowering begins. The early flowering stage of the peanut is the critical moment to apply biocontrol agents. Temperature-sensitive hydrogel beads containing kaolin could be considered as carriers of biocontrol agents for the control of aflatoxin in peanuts.

## 1. Introduction

Aflatoxin contamination of peanuts, caused by the invasion of toxigenic strains of *Aspergillus flavus* and *Aspergillus parasiticus*, has attracted extensive attention. The peanut plant has the characteristics of aboveground blooming and underground fruiting. The pods are in direct contact with soil fungi, and soil is the main source of mold that infects peanuts [1]. Biological control methods for reducing the amount of aflatoxin using non-aflatoxigenic strains to inhibit toxin-producing strains have proved to be effective in agriculture [2,3].

Previous studies have shown that sterilized grains such as rice and barley can be used as biocontrol carriers [3,4]. These starch-rich carriers provide favorable conditions for the growth and sporulation of fungi after absorbing water. In recent decades, an increasing number of bioplastic-based products have been used in the field of biocontrol [5]. A bioplastic mainly composed of modified starch has been studied to verify the possibility of replacing grains [6]. The bioplastic granules act as carriers of biocontrol fungus, in which starch can provide the carbon source. A sprayable bioplastic formulation prepared from inoculated recycled bioplastic has been verified to be effective at delivering biocontrol fungus to corn [7].

Natural polysaccharides have attracted widespread interest as materials for encapsulation and controlled release [8]. Starch is renewable, degradable, and inexpensive, and it can be used to improve the dispersion and stability of microcapsules [9,10,11]. Alginate is a polysaccharide produced by brown macroalgae that has excellent biodegradability, biocompatibility, porosity, and hydrophilicity [12]. Alginate is usually used as an entrapment formulation after crosslinking with metal ions. Controlled-release systems can decrease the release rate of entrapped substances, reduce the amount of leakage, and allow the entrapped substances to last longer in the environment. Starch–alginate formulations have been demonstrated to be utilized in the encapsulation of proteins [13], antioxidants [14], biocontrol *Trichoderma* [15], and the controlled release of agrochemicals such as thiram [16], soproturon, and imidacloprid [17]. In a previous work, we developed starch–alginate-based formulations for the simultaneous encapsulation of biocontrol fungus and metalaxyl, and the release rate was observed to slow down with the addition of kaolin and rice husk powder [18]. The release rate of spores and metalaxyl decreased with the increase of the concentration of the additives. There have been many reports on the encapsulation and sustained release of microorganisms or agrochemicals by polysaccharides, but most of these formulations were diffusion or volatilization controlled, which makes it difficult for them to respond to environmental stimuli.

Smart hydrogels, which can undergo abrupt changes or phase transitions when encountering external stimulation, such as temperature, pH, and light, have attracted considerable attention [19,20,21]. Among these environmental factors, temperature is important because it is easy to control and plays an important role in crop growth. Temperature-sensitive hydrogels have comprehensive applications in the controlled release of drugs, tissue culture, and the immobilization of protein [22,23,24]. In agriculture, temperature-responsive polymers have been investigated as pesticide carriers, as the release rate can be controlled to meet the requirements of pest prevention [25,26]. Poly(*N*-isopropylacrylamide) (PNIPAAm) is a typical temperature-responsive polymer, exhibiting a temperature-dependent volume phase transition near the lower critical solution temperature (LCST) in aqueous solution [27,28]. As PNIPAAm chains contain both hydrophilic and hydrophobic groups, PNIPAAm gel exhibits thermoreversible phase separation upon the temperature cycle across the LCST [29]. At temperatures below the LCST, the gel forms a homogeneous system in aqueous media due to the hydrogen bonds between water molecules and amide side chains. At temperatures above the LCST, the PNIPAAm chains assume a collapsed conformation and separate from the water phase; meanwhile, the loaded substances are also expelled from the gel interior [21]. Temperature-responsive hydrogel microspheres composed of alginate and PNIPAAm can be formed by semi- or full interpenetrating network (IPN) methods [30,31,32]. These polymers have been well developed in the field of smart gel materials. Shi et al. encapsulated indomethacin in an alginate–PNIPAAm hydrogel and was released quickly when the temperature was 37 °C above the LCST [33]. Juliana F. Piai and coworkers investigated the release characteristics of bovine serum albumin (BSA) from an alginate-Ca^2+^/PNIPAAm hydrogel with thermosensitivity and established a release kinetic model [34]. While these materials have been used for the controlled release of drugs, their applications in microorganisms or agriculture are scarce.

PNIPAAm gel is known to be thermoresponsive at an LCST of 32 °C [35]. IPN hydrogels containing PNIPAAm usually have temperature sensitivity near 32 °C. Shi et al. measured the LCST of alginate beads containing PNIPAAm and found that it was between 28.6 and 29.6 °C [36]. The LCST of IPN hydrogel composed of PNIPAAm and sericin was determined to be approximately 30 °C [37]. Previous studies on biological control demonstrated that the application of biocontrol agents after 50 days of peanut cultivation was effective [38]. The temperature at which the peanuts grow during this time is about 30 °C. In that case, thermosensitive beads containing PNIPAAm can release biocontrol agents quickly after the temperature reaches the LCST, thereby achieving better biological control effects.

This work aimed to develop a formulation to achieve the temperature-triggered release of biocontrol agents at a suitable time, thereby achieving biological control of aflatoxin. Semi-IPN hydrogel beads based on starch, alginate, and PNIPAAm were prepared for the delivery of biocontrol fungus spores. The ingredients in the carrier were biocompatible and biodegradable and served as the carbon source for the growth and sporulation of the biocontrol agents after moisture adsorption. To the best of our knowledge, this is the first example of the use of a temperature-responsive bead as an intelligent carrier for the controlled release of biocontrol agents aimed at aflatoxin contamination. The swelling behavior of these semi-IPN beads was investigated as a function of temperature, pH, and ionic strength. Fourier-transform infrared spectroscopy (FTIR), thermogravimetry analysis (TGA), differential scanning calorimetry (DSC), scanning electron microscopy (SEM), and rheological analysis were used to characterized the beads with different formulations, and the temperature-triggered release behavior of the biocontrol agents was examined. 

## 2. Experimental Section

### 2.1. Materials

*N*-isopropylacrylamide (NIPAAm), soluble starch (maize starch), Tween 20, and sodium alginate (SA) were obtained from Aladdin Biochemical Technology (Shanghai, China). The viscosity of SA (1 wt %, 20 °C) was 200 ± 20 mPa∙s. Ammonium persulfate (APS) and *N*,*N*,*N*ʹ,*N*ʹ-tetramethylethylenediamine (TEMED) were supplied by Shanghai Chemical Reagent Co., Ltd. (Shanghai, China). Calcium chloride, potassium bromide, and phosphate buffer solution (PBS) were purchased from Sino Pharm Chemical Reagent Co., Ltd. (Shanghai, China). Dichloran 18% glycerol (DG18) agar was provided by Beijing Aoboxing biotech Co., Ltd. Kaolin was purchased from Xincheng Fine Chemical Co., Ltd. (Shanghai, China). Nontoxic *A. flavus* strains (H 4–5) were isolated from our laboratory in Changchun, China. Ultrapure water was used in the experiment. All chemicals were used without further purification. 

### 2.2. Preparation of Thermosensitive Hydrogel Beads

#### 2.2.1. Preparation of Spore Suspensions

The isolated nontoxic strains were grown and maintained on DG18 agar. After 2 weeks of incubation at 30 °C in the dark, the spores were removed by lightly scraping the medium and were suspended in aqueous 0.2% Tween 20. The density of the spore suspensions was determined by a hemocytometer and adjusted as needed [39].

#### 2.2.2. Synthesis of PNIPAAm

PNIPAAm was obtained through radical polymerization using NIPAAm as a monomer, APS as an initiator, and TEMED as a catalyst. Specifically, an aqueous solution was prepared by dissolving 35.3 mmol of NIPAAm and 1.35 mmol of APS in 45 mL of water. Then, nitrogen was purged into the solution for 30 min to remove oxygen. After that, 0.267 mmol of TEMED was added. The polymerization lasted for 6 h at room temperature (20 °C) [21]. After the reaction was completed, the reactants were precipitated in hot water and then dissolved in distilled water. The product was vacuum dried at 40 °C for 24 h.

#### 2.2.3. Preparation of Thermosensitive Hydrogel Beads

The quantitative starch, alginate, and kaolin were dissolved in hot water by magnetic stirring (Table 1). After forming a homogeneous solution, the aqueous solution was evenly mixed with PNIPAAm in a certain ratio, and then 10% *v*/*v* spore suspensions were added. After stirring for 30 min until becoming uniform, the dispersion was added dropwise by a 50 mL syringe (needle diameter of 1.2 mm), from a 30 cm height, into 100 mL of 2% *w*/*v* CaCl_2_ solution under constant stirring. Thus, the spherical beads were formed and maintained in the CaCl_2_ solution until completely crosslinked. After that, the beads were washed with ultrapure water to remove excess ions, air-dried overnight, and then vacuum-dried to constant weight at 40 °C. The characteristics of the beads are shown in Table 2.

### 2.3. Characterization

The molecular weight (Mn) and polydispersity (PDI) of the synthesized PNIPAAm were measured by a Waters 1525 gel permeation chromatograph (GPC) (Milford, MA, USA) equipped with microstyragel columns and a 2414 differential refractometer detector. The eluant was tetrahydrofuran (THF), and the flow rate was 1 mL/min. The calibration curve was obtained using monodisperse polystyrene as the standard.

Thirty thoroughly dried beads were taken from each formulation, and their diameters were determined by a Vernier caliper. FTIR, SEM, and TGA were used to characterize the different formulations.

The FTIR spectra of the samples were measured using a Nexus670 FTIR spectrometer (Thermo Nicolet Corporation, Madison, WI, USA) by the conventional KBr disk tablet method.

TGA was performed using a SHIMADZU DTG-60H thermal analyzer (SHIMADZU Corporation, Kyoto, Japan) at a heating rate of 10 °C/min in the range of 20–700 °C under a nitrogen atmosphere, with a flow rate of 50 mL/min.

The surface morphology of the hydrogels was observed by SEM using a HITACHI S-4800 (Hitachi Limited, Tokyo, Japan). Samples were dissolved in ultrapure water at room temperature, quickly frozen in liquid nitrogen, and then freeze-dried (Beijing Boyikang Laboratory Instruments Co., Ltd., Beijing, China) for 48 h. The lyophilized hydrogels were fixed on a copper plate and sputtered with gold to avoid charging effects.

### 2.4. Determination of Thermosensitive Hydrogel Temperature-Sensitive Behavior and LCST

DSC measurements (DSC-7, Perkin-Elmer, Waltham, MA, USA) were used to determine the LCST of the semi-IPN hydrogel beads. First, all of the beads were swollen in PBS for 24 h at room temperature. Then, DSC analysis of the swollen beads was carried out from 20 to 40 °C at a rate of 1 °C/min and under a nitrogen flow rate of 20 mL/min. Temperature and heat flow were calibrated using a pure indium standard at the same ramp rate.

The absorbance of the semi-IPN hydrogels at 480 nm was determined using a UV–vis spectrophotometer (SHIMADZU Corporation, Kyoto, Japan). The hydrogels were placed in a cuvette in a temperature-controlled box. The temperature was raised from 25 to 40 °C, and each sample was allowed to stand for 15 min before measuring the absorbance.

### 2.5. Swelling Studies

The swelling properties of the samples were measured gravimetrically. The weighed dried beads were immersed in PBS (pH 6.0) separately at 25, 30, and 35 °C. After 12 h, the swollen beads were taken out and weighed after removing the excess water on the surfaces with filter paper. The swelling ratio (SR) was obtained by the following expression:

(1) SR=(Ws-Wd)/Wd

where *Ws* and *Wd* are the weight of the swollen and dried beads, respectively. The experiment was repeated three times for each group.

To investigate the ionic strength dependence of the swelling of the beads, the quantitative beads were placed in 20 mL of NaCl aqueous solutions at different ionic strength values (0.1 ≤ I/mol L^−1^ ≤ 0.8) at 25 °C. The effect of pH on the swelling properties of the beads was studied by varying the pH of the medium. The weighed dried beads were immersed in 20 mL of PBS (pH 5.0, 5.5, 6.0, 6.5, and 7.0). The method of obtaining the swelling ratio was the same as the above-described method.

### 2.6. Rheological Properties

The rheological properties of the samples were determined in aqueous solutions using a dynamic shear rheometer (AR500, TA Instruments, Fort Sam Houston, TX, USA). The aqueous samples prior to crosslinking were gently placed on the surface of the lower Peltier plate, and the upper plate declined until it reached a 1000 μm gap distance. A solvent trap was used to minimize evaporation during all measurements. Temperature sweep experiments from 25 to 40 °C were carried out at a scan rate of 1 °C/min. The shear rate (10 s^−1^) and test time (1 min) were the same for all measurements.

### 2.7. Release Measurements

#### 2.7.1. Entrapment Efficiency of Spores

The colony-forming unit (CFU/g) method was used to evaluated the entrapment efficiency of the spores. Specifically, 0.1 g of beads of each formulation was dissolved in 10 mL of sodium citrate solution (2.0% *w*/*v*) and then gradient diluted in 0.85% *w*/*v* sodium chloride solution. On DG18 agar, 100 μL of aliquots was inoculated for 3–5 days at 28 ± 2 °C. The results are expressed in terms of the colony-forming units per gram of beads. The quantity of spores released after the dissolution of beads divided by the total number of spores applied was the entrapment efficiency of spores.

#### 2.7.2. Release Properties of Spores

The release characteristics of the semi-IPN beads were investigated by immersing the prepared beads of each formulation (1 g) in 50 mL of PBS. The pH of the PBS was adjusted to 6.0 to be consistent with that of peanut soil. The solution media were placed in the dark and maintained at 25, 30, and 35 °C. The density of the spores in the release medium was determined by plate counting. The sample (200 μL) was periodically taken out and serially diluted in PBS before plating. Aliquots of 100 μL of each gradient concentration were inoculated on DG18 agar for 3–5 days at 28 ± 2 °C, and the number of colonies of *A. flavus* was recorded. These experiments were typically conducted in triplicate.

#### 2.7.3. Release Kinetics of Spores

The release kinetics of the spores from the beads was analyzed by an empirical model according to the cumulative release data versus time [40,41].

(2) $$\frac{M_{t}}{M_{0}}=kt^{n}$$

where $M_{t}/M_{0}$ is the fraction release of spores in time *t*, *k* is the release rate constant, and *n* is the diffusion exponent of the release system. In terms of the release mechanism, for normal Fickian diffusion, *n* = 0.5; for case II diffusion, *n* = 1.0; and for non-Fickian, *n* = 0.5–1.0.

### 2.8. Statistical Analysis

Triplicate experiments were performed, results are expressed as mean ± standard deviation (SD), and significance was analyzed using Duncan’s multiple comparison tests in SPSS Statistics 20.0 (IBM Corporation, NY, USA). The criterion for significance was *p* < 0.05.

## 3. Results and Discussion

### 3.1. Effect of Formulation Parameters on the Characteristics of the Beads

The characterization of the synthesized PNIPAAm was obtained from the GPC test, and the data showed that the average molecular weight and PDI were 26,156 and 1.8, respectively. The composition of the sample beads is shown in Table 1. Most of the sample beads were spherical in shape, and their diameter ranged from 1.93 ± 0.08 to 2.55 ± 0.13 mm in different formulations. Spores were entrapped in the beads, and the entrapment efficiency of the different formulations varied from 43.25 ± 1.88 to 90.48 ± 1.53 (Table 2). It was found that the effect of different formulations on the entrapment efficiency was significant (*p* < 0.05). Similar results have been previously reported [18].

### 3.2. Thermosensitive Behavior of Semi-IPN Hydrogels

**Figure 1:**
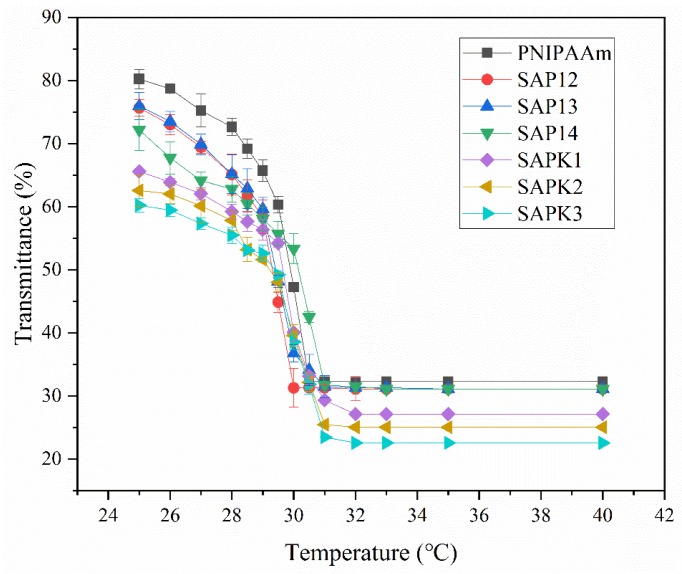
Temperature dependence of transmittance of semi-interpenetrating network (IPN) hydrogels.

PNIPAAm hydrogels undergo a phase change near the LCST in aqueous solution, and the phase state of semi-IPN hydrogels at different temperatures can be expressed by the transmittance of light. The changes in transmittance with rising temperature were recorded from 25 to 40 °C using a UV–vis spectrophotometer, as shown in Figure 1. Initially, the transmissivity of the semi-IPN hydrogels gradually decreased with increasing temperature. However, there was a rapid decrease in transmittance around 29–30 °C for these samples. The temperature corresponding to the point where the temperature–transmittance curve dropped fastest was the LCST. The transmittance results showed that the LCST of pure PNIPAAm was around 29.5 °C, and the difference between different formulations was not obvious. From this, it can be determined that the LCST of the semi-IPN hydrogel samples ranged from 29 to 30 °C, which is similar to the range already reported for the LCST of PNIPAAm [37,42] and consistent with the DSC data.

### 3.3. Characterization

#### 3.3.1. FTIR

**Figure 2:**
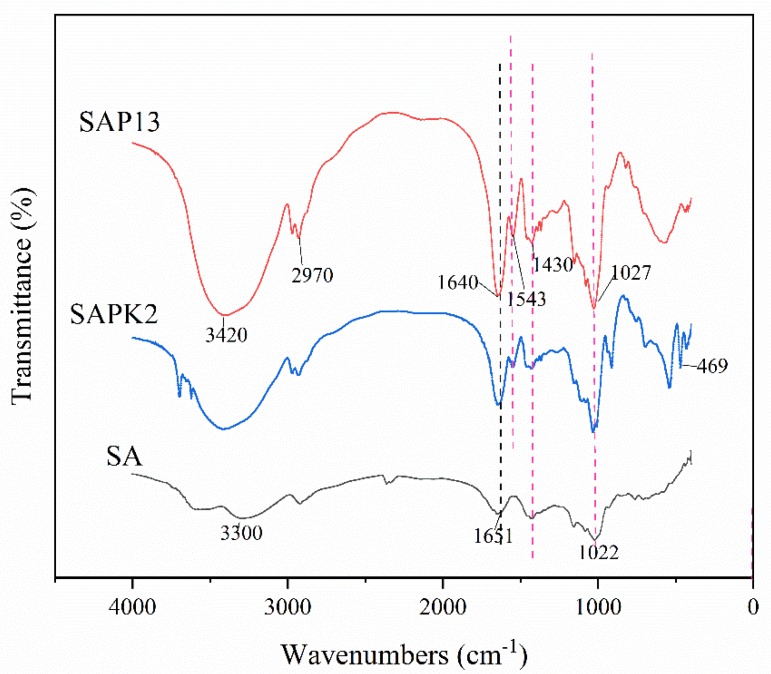
FTIR spectra of hydrogel beads.

The results of the FTIR analysis of SA, SAP13, and SAPK2 are presented in Figure 2. It is obvious that in all analyzed spectra, a broad peak around 3400 cm^−1^ was present owing to O-H stretching vibrations. This broad peak may come from hydroxyl groups in G and M residues of the alginate chain [43]. The absorption band at 2970 cm^−1^ of SAP13 and SAPK2 corresponded to the stretching of CH_3_ groups in PNIPAAm. The asymmetric stretching vibration of COO^–^ groups in the alginate network was manifested at the peak near 1640 cm^−1^ [44]. The peak at 1543 cm^−1^ of SAP13 and SAPK2 could be assigned to the N–H bending of the amide group, which was related to PNIPAAm [45], and this peak did not appear on the SA spectra. In all three curves, the band at around 1027 cm^−1^ was due to the C–O stretching of alginate structural units [46]. In particular, it was observed that the peak at 469 cm^−1^ of SAPK2 was due to the Si–O bending modes that belonged to kaolin [47].

#### 3.3.2. Thermogravimetric Analysis

**Figure 3:**
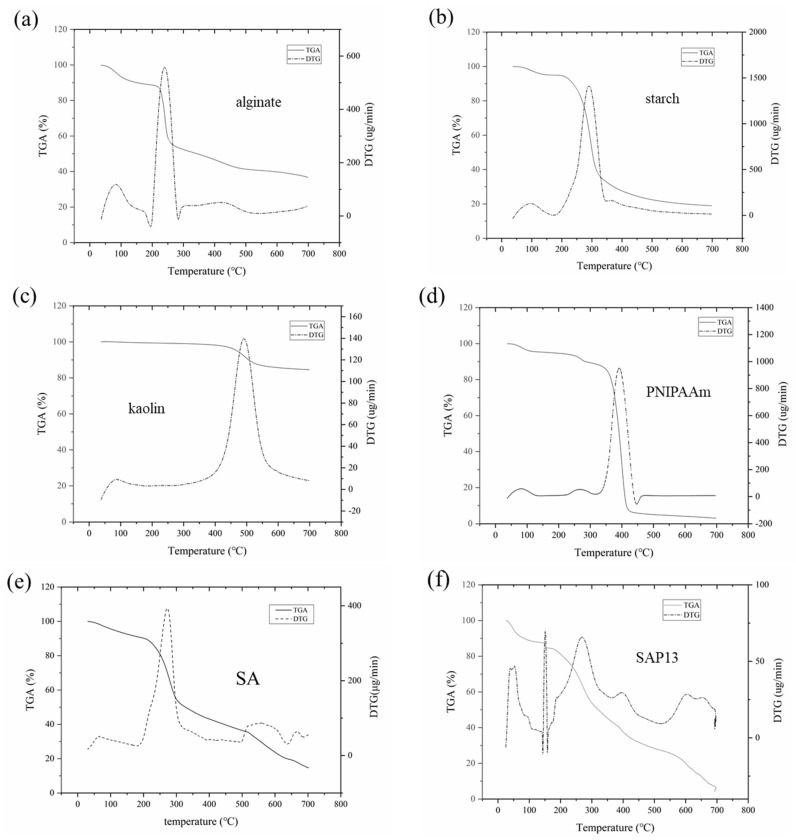

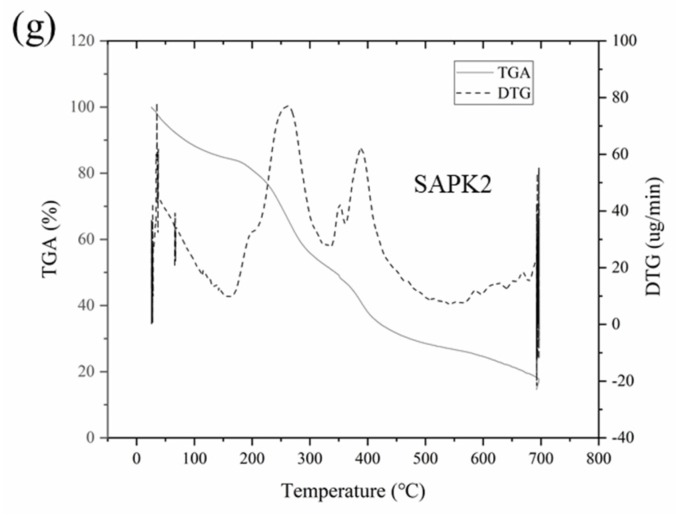
Thermogram of pure components and hydrogel beads: (**a**) alginate, (**b**) starch (**c**), kaolin, (**d**) PNIPAAm, (**e**) SA, (**f**) SAP13, and (**g**) SAPK2.

The thermal characteristics of the samples were investigated by thermogravimetry. The TGA and derivative thermogravimetry (DTG) curves of pure components (alginate, starch, PNIPAAm, and kaolin) and hydrogel samples with various compositions, obtained at a heating rate of 10 °C min^−1^, are shown in Figure 3. The thermal behavior of the degradation of the starch–alginate formulation and the semi-IPN hydrogel beads was studied in order to better understand the thermal degradation characteristics of the gel.

Normally, thermal decomposition entails moisture loss, dehydration, and decomposition processes [48]. The dehydration process of the samples, occurring within the range 25–150 °C, could be assigned to the absorbed water and structural water deficiency, which accounted for about 10–15% of the weight loss. However, the weight of kaolin was almost unchanged in this process.

According to the thermogravimetric analysis of the pure components, the temperatures of the maximum rate of mass loss were 240, 297, 498, and 401 °C for alginate, starch, kaolin, and PNIPAAm, respectively. Correspondingly, the temperatures of the maximum rate of mass loss of SA, SAP13, and SAPK2 hydrogel beads were 252, 275, and 301 °C. The results showed that the temperatures of the maximum rate of weight loss of PNIPAAm and kaolin were much higher than those of alginate and starch. Consequently, SAPK2 had a higher decomposition temperature than SAP13, and SAP13 had a higher decomposition temperature than SA.

Compared with the SA hydrogel, the DTG curves of SAP13 and SAPK2 hydrogels were more complicated due to there being more interactions between the components. For all samples, the remaining residues at the end were 36.78%, 18.89%, 84.54%, 2.97%, 19.32%, 4.89%, and 14.57%, for alginate, starch, kaolin, PNIPAAm, SA, SAP13, and SAPK2, respectively. Compared with SAP13, it was obvious that there was more residue of the hydrogel containing kaolin (SAPK2), which was due to the presence of oxides, carbonates, and silicates [47]. In addition, the initial decomposition temperatures were 151.38 and 182.43 °C for SAP13 and SAPK2. The results indicate that, with the addition of kaolin, the thermal stability was improved with respect to the hydrogel beads. This finding is consistent with the extant research [49,50].

#### 3.3.3. SEM

**Figure 4:**
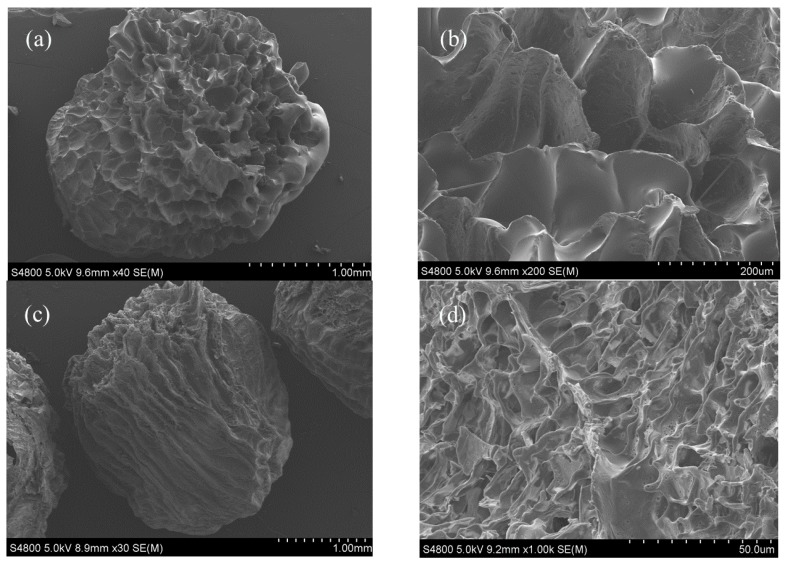

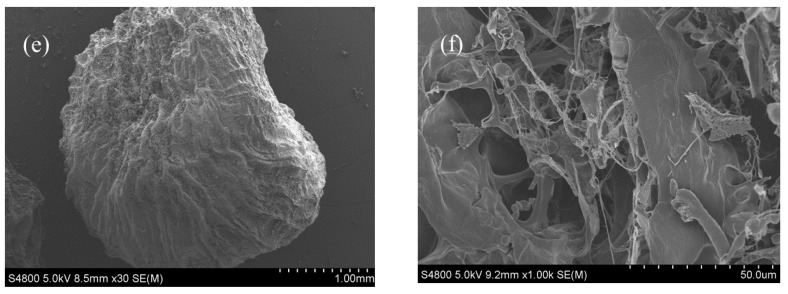
SEM of hydrogel beads: (**a**,**b**) SA beads at different magnifications ( ×40, ×200). (**c**,**d**) SAP13 beads at different magnifications (×30, ×1000). (**e**,**f**) SAPK2 beads at different magnifications (×30, ×1000).

Figure 4 shows the SEM micrographs of the investigated hydrogel beads. The porous structure of the hydrogel beads was preserved by the lyophilization. It was observed that the studied hydrogel beads had a spherical shape (at 30× magnification), which became an oval shape with the addition of kaolin. This phenomenon may have been caused by the decrease of viscosity, which can be proved by the results of rheological property analysis. Similar results have been reported in a previous study [47].

At high magnification (1000× magnification), the honeycomb structure on the surface of SAP13 and SAPK2 could be noticed. Dumitriu’s research revealed a porous morphology with a honeycomb-like structure of PNIPAAm/alginate hydrogels crosslinked with N, N’-methylenebisacrylamide (BIS), which is consistent with our results [32]. In contrast, there was no such structure on SA, which was flatter. In particular, kaolin was interspersed in SAPK2’s honeycomb structure, which made the SAPK2 surface less regular than that of SAP13. These structural differences affect the release properties of the entrapped objects.

#### 3.3.4. Swelling Studies

**Figure 5:**
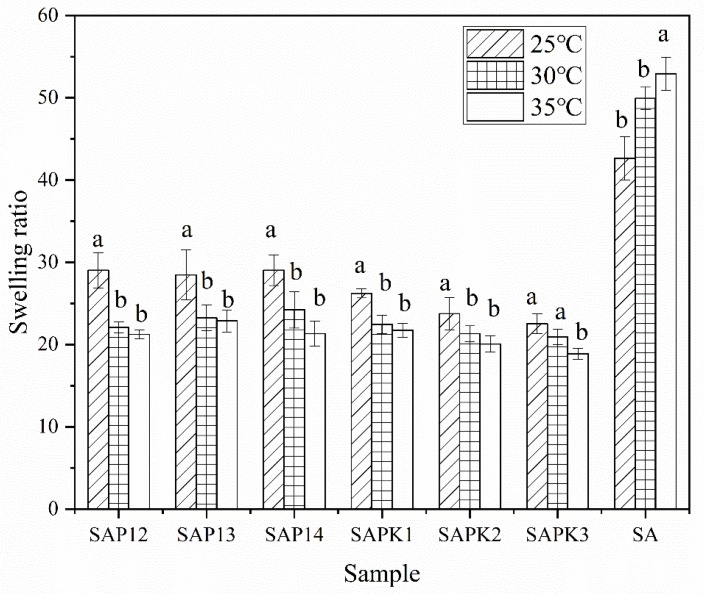
Swelling ratio of beads with different compositions at different temperatures (different letters in the graph indicate significant differences in swelling ratio of the beads at different temperatures using Duncan’s multiple tests).

Figure 5 displays the swelling degree of the beads prepared at different temperatures. After the beads were immersed in the buffer solution of pH 6.0, the carboxylic acid groups in the solution became ionized, and a small amount of hydrogen ions in the water acted as a bridge among the alginate; then, the beads absorbed water and swelled.

As is shown in Figure 5, compared with the starch alginate formulation, the swelling ratio of the semi-IPN hydrogel beads containing PNIPAAm decreased considerably. This may be due to the formation of more hydrogen bonds in the starch alginate beads, resulting in a higher degree of swelling. Figure 5 also indicates that the swelling ratio tended to decrease in PBS as the temperature increased for all semi-IPN hydrogel beads. For instance, the swelling ratio of SAP13 was 28.47 at 25 °C and reached 19.09 at 35 °C. Similar results have been observed by J.F. Mano and Yang Liu [51,52]. This phenomenon is attributed to the shrinkage of the PNIPAAm chains when the temperature exceeds the LCST of PNIPAAm. However, the swelling ratio of the starch–alginate beads increased with the increase of temperature. This was due to the rise of temperature, which increased the ion exchange rate in the solution, thus increasing the swelling ratio.

A statistical analysis was performed to analyze the effect of the composition on the swelling ratio, which is shown in Table 3. The results showed that the difference between different formulations was significant. In particular, the incorporation of kaolin reduced the swelling ratio of the beads significantly, as kaolin can be considered to act as a crosslinking agent during polymerization and decreases the water absorbency of the beads [53].

**Figure 6:**
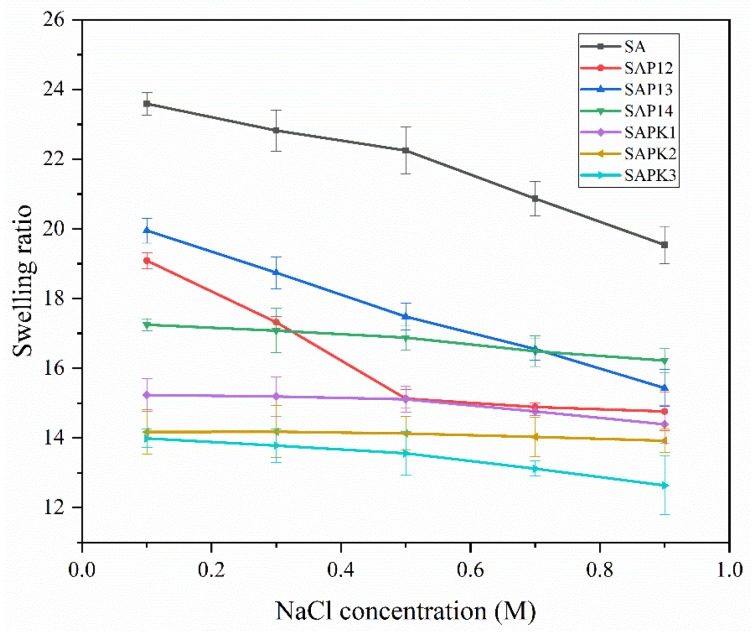
Effect of ionic strength on the swelling behaviors of beads with different compositions.

The effect of ionic strength on the swelling behavior of the beads was investigated in NaCl aqueous solutions with a range of 0.1–0.9 mol L^−1^ at pH 7.0 and room temperature. As shown in Figure 6, the swelling ratio of all samples decreased with the increase of ionic strength. One reason for this was the difference in osmotic pressure between the hydrogel and the aqueous phase. Another reason was that the charge screening effect or shielding effect could influence the swelling capacity, as anion–anion repulsion was prevented by cations [54]. Similar results regarding the ionic strength dependence of hydrogels have been reported by others [55,56].

**Figure 7:**
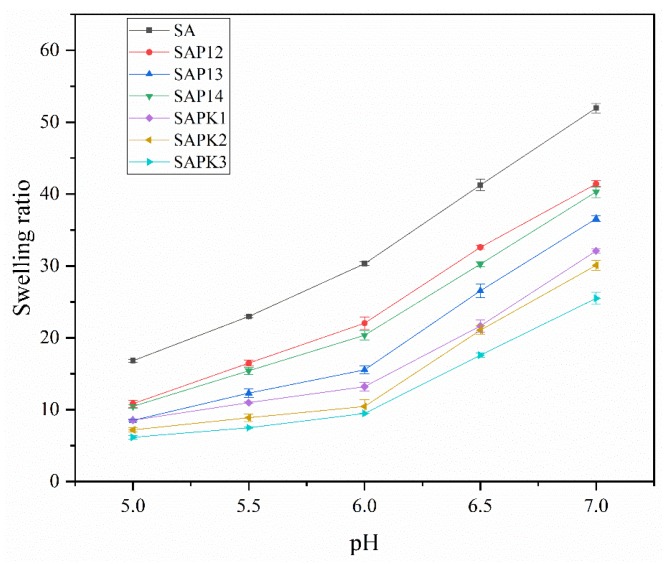
pH-dependent swelling behaviors of beads with different compositions.

The pH dependence of the swelling behavior of the beads was studied at room temperature at various pH values between 5.0 and 7.0 (Figure 7). The obtained results showed that the swelling of beads of all formulations increased as the pH increased from 5.0 to 7.0. As all formulations were based on alginate, all of them were pH sensitive. The –COOH groups were converted to –COO^–^ at pH > 3.2, and this could cause high anion–anion repulsion, which in turn resulted in a higher swelling capacity. This type of research was also studied by others, who had similar findings [31,33,54].

### 3.4. Rheological Properties

**Figure 8:**
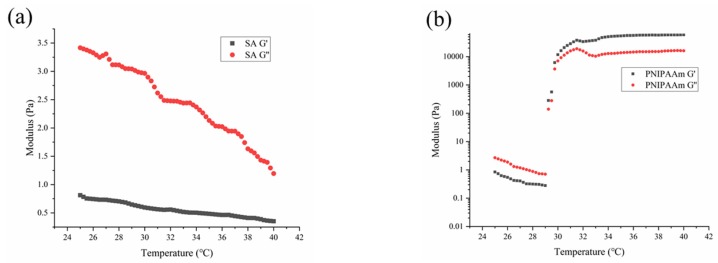

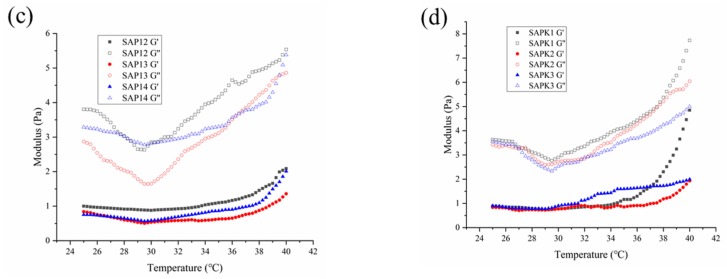
Temperature dependence of storage modulus (G’) and loss modulus (G’’) of aqueous samples prior to crosslinking: (**a**) SA, (**b**) PNIPAAm, (**c**) SAP12, SAP13, and SAP14, and (**d**) SAPK1, SAPK2, and SAPK3.

The rheological properties of the samples were investigated by monitoring the storage modulus (G’) and loss modulus (G’’) as a function of temperature (Figure 8). The G’’ values were obviously larger than the corresponding G’ values within the temperature range of 25–40 °C (Figure 8a), showing typical liquid viscosity behavior, and the storage and loss moduli gradually decreased with the temperature for the SA formulation, which exhibited Arrhenius behavior [57]. Figure 8b indicates that there were two processes involved in the change of the G’ and G’’ of PNIPAAm as the temperature increased. In the first phase, viscoelastic properties exhibited an initial behavior governed by the Arrhenius law, where G’ and G’’ decreased with temperature. Then, the slope of modulus versus temperature became positive, which meant that the PNIPAAm hydrogels started to exhibit their hydrophobicity as a consequence of the conformational coil–globule transition related to the LCST. Furthermore, G’ increased faster than G’’, and G’ values became much larger than G’’, showing elastic behavior, concerning the sol–gel transition [58]. As is shown in Figure 8c,d, the modulus law of the aqueous samples containing PNIPAAm changed with temperature and was similar to that of PNIPAAm. First, the G’ and G’’ decreased as the temperature increased, and then it started to increase after reaching a critical temperature. The observed critical temperature was consistent with the previously characterized LCST results. However, within the temperature range of 25–40 °C, there was no case where G’ values were larger than G’’, which was due to the presence of starch and alginate.

Considered together, the transmittance and viscosity results indicate that the formulations containing PNIPAAm were stable at normal temperature but dissociated above the LCST (close to the temperature of the flowering stage of peanuts). As such, these formulations may be suitable carriers for biocontrol agents.

### 3.5. Release Properties of Spores from Thermosensitive Hydrogel Beads

**Figure 9:**
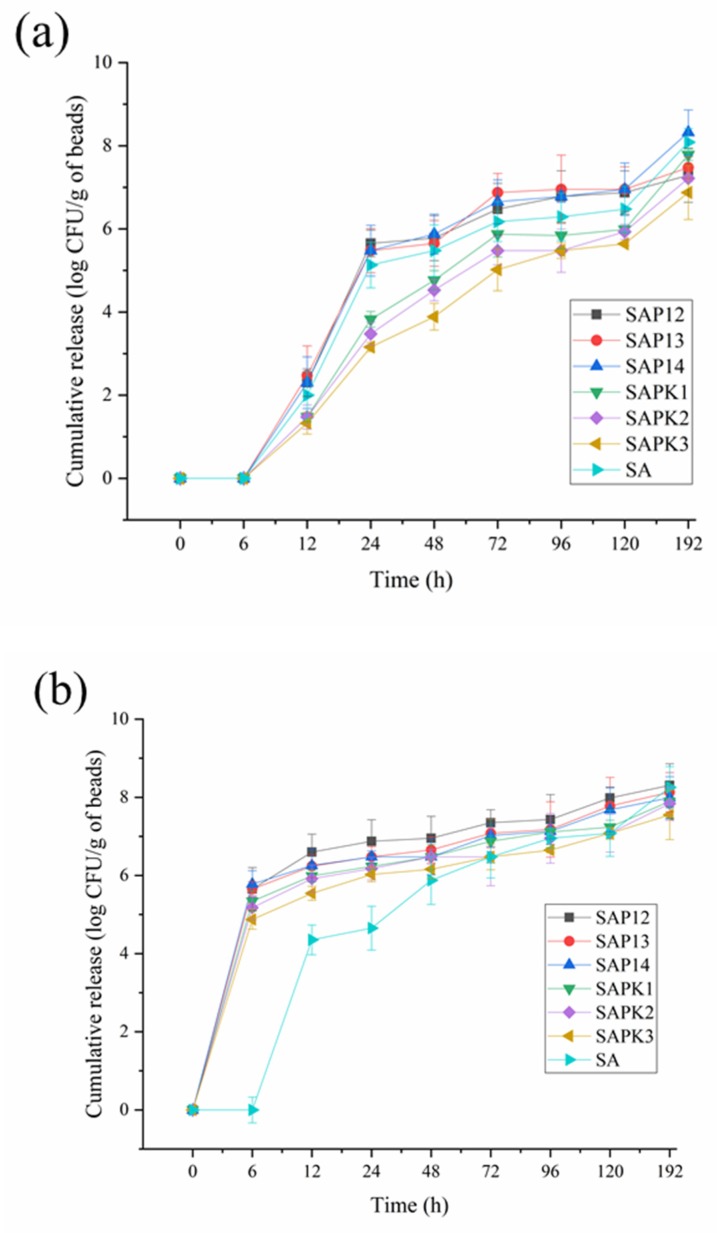

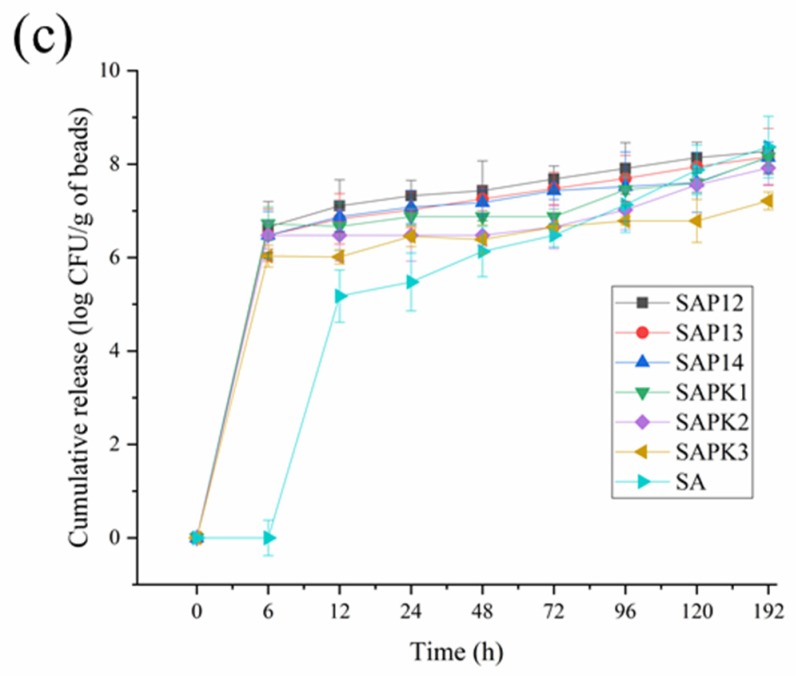
Release profiles of the spores from the studied beads (**a**) at 25 °C, (**b**) at 30 °C, and (**c**) at 35 °C.

The release of biocontrol agents from the beads requires two processes. Solvents penetrate the network of polymers and swell, then the biocontrol agents diffuse along the aqueous pathways to the outside of the beads. In this work, the dried and loaded samples were immersed in PBS (pH 6.0) at 25, 30, and 35 °C in the dark to simulate the environment of peanut growth. The release profiles are shown in Figure 9. 

**Figure 10:**
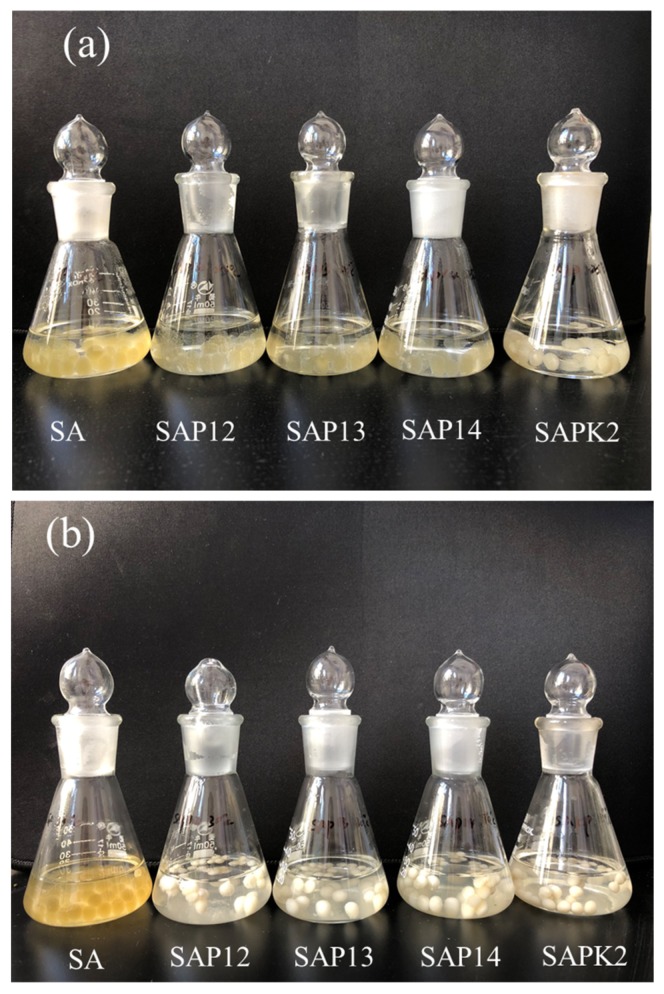
Photograph of hydrogel beads with different compositions (**a**) at 25 °C and (**b**) at 30 °C. The state of beads at 35 °C was close to that at 30 °C.

As is shown in Figure 9a, at 25 °C, no released spores were detected after 6 h, and all samples showed sustained release behavior over time. When the temperature of the release medium rose to 30 °C (Figure 9b), which is around the LCST of the semi-IPN hydrogel, the release rate of the semi-IPN hydrogel beads was much higher than that of the starch–alginate sample. As can be seen from Figure 10, the color of all the beads changed significantly at 30 °C compared with 25 °C, except for the SA beads. This was due to the fact that PNIPAAm produced a white precipitate at temperatures above the LCST. The PNIPAAm chains became more hydrophobic and collapsed at temperatures above 30 °C, which led to larger pore sizes of the gel, and this could accelerate the diffusion of the medium into the beads, thus speeding up the release rate [59]. Shi et al. investigated the release behavior of indomethacin from calcium alginate/PNIPAAm beads. The results showed that the release rate of the drug was higher at 37 °C than that at 25 °C, and the determined LCST of the samples was around 33.5 °C [33]. Similar results have been reported by Shi. J and Sun. X [21,60].

According to Figure 9b,c, when the temperature was above (including) 30 °C, after the beads swelled in the release medium, the semi-IPN hydrogel samples containing PNIPAAm exhibited an abrupt release after 6 h. The number of released spores from SAP13 beads could reach 5.65 log CFU·g^−1^; when the temperature rose to 35 °C, the number was higher. In comparison, there were no spores released from SA beads at the same time. Moreover, the addition of kaolin could obviously delay the release of spores from the semi-IPN hydrogel beads at 25 °C. The number of spores released from SAPK1, SAPK2, and SAPK3 beads was an order of magnitude lower than that released from SAP13 within 12–120 h. The retarded release with the increasing kaolin content may have been due to better interaction between kaolin and other composites [47]. Results correspond to the swelling behavior of these formulations. This phenomenon is consistent with the results of our previous work [18]. He. Y and colleagues encapsulated *Raoultella planticola* Rs-2 in alginate-based formulations containing clay. They found that the swelling and release rate decreased with increasing bentonite content, which can be attributed to the strong interactions between bentonite and sodium alginate [61]. In addition, the release behavior of SAPK1, SAPK2, and SAPK3 at temperatures above the LCST indicated that the formulation with kaolin had good thermoresponsive properties. Li’s study showed that IPN hydrogels containing clay exhibited temperature sensitivity, and the results implied that the temperature sensitivity could be weakened by increasing the clay content owing to the steric hindrance of clay platelets [62]. Furthermore, Figure 9 shows that the release rate increased with the increase of temperature for all beads. This may have been associated with the swelling properties of alginate.

As for the release mechanism, the release kinetic constant *k* and the diffusional exponent *n* were evaluated from the slope and intercept of the plot of ln (*M_t_*/*M*_0_) versus ln *t*. The values of *n* and *k* are shown in Table 4, which suggest that the release mechanism of all beads was normal Fickian at different temperatures, which is similar to the results of our previous study [18].

The release profile of the samples showed that the release of spores from the semi-IPN hydrogel beads was sustainable and controllable with good thermosensitivity. Particularly, the addition of kaolin could meet the characteristics of early application of biocontrol agents and burst release after the temperature was above 30 °C, which is close to the temperature of peanut flowering.

## 4. Conclusions

Thermosensitive hydrogel beads were successfully prepared by combining PNIPAAm with starch–alginate hydrogel. The biocontrol agents of non-aflatoxigenic spores were entrapped in the beads, as confirmed by the determination of entrapment efficiency. The morphology of the semi-IPN hydrogel showed a porous honeycomb structure on the surface of the beads. The measurement of thermosensitive behavior and rheological properties indicated that the semi-IPN hydrogel demonstrated an obvious phase transformation around 29–30 °C, and this LCST is close to the initial temperature of peanut flowering. The temperature change at flowering was identified as the main trigger mechanism for hydrogel dissolution and biocontrol agent release. The beads based on the semi-IPN hydrogel with kaolin could serve as carriers of biocontrol agents. 

## Figures and Tables

**Table 1:** Formulation parameters for the synthesis of hydrogel beads.

Sample	PNIPAAm/Alginate	CaCl_2_ Solution Concentration	Starch	Alginate	Kaolin
	(*v*/*v*)	(% *w*/*v*)	(% *w*/*v*)	(% *w*/*v*)	(% *w*/*v*)
SA	/	2	10	1.5	/
SAP12	1:2	2	10	1.5	/
SAP13	1:3	2	10	1.5	/
SAP14	1:4	2	10	1.5	/
SAPK1	1:3	2	10	1.5	1
SAPK2	1:3	2	10	1.5	2
SAPK3	1:3	2	10	1.5	3

**Table 2:** Characteristics of the hydrogel beads.

Sample	Beads Weight (g)	Average Bead Diameter (mm)	Encapsulation Efficiency (%)	LCST (°C)
SA	2.13	1.93 ± 0.08 ^c^	48.25 ± 3.67 ^d^	/
SAP12	2.81	2.55 ± 0.13 ^a,b,^*	67.44 ± 4.36 ^c^	29.2
SAP13	2.38	2.41 ± 0.05 ^a,b^	66.65 ± 2.94 ^c^	29.3
SAP14	2.49	2.33 ± 0.18 ^b^	43.25 ± 1.88 ^e^	30.0
SAPK1	2.68	2.46 ± 0.16 ^a,b^	64.36 ± 2.16 ^c^	29.7
SAPK2	2.95	2.54 ± 0.21 ^a,b^	90.48 ± 1.53 ^a^	29.5
SAPK3	3.11	2.63 ± 0.12 ^a^	75.32 ± 2.22 ^b^	29.3

* Mean ± standard deviation with different letters represent for significant differences between formulations by Duncan’s multiple range tests, “^a, b, c, d, e^” different letters in the same series indicate significant difference at *p* < 0.05.

**Table 3:** Swelling ratio of beads with different compositions at different temperatures (the marked letters indicate significant differences in swelling ratio of the beads with different compositions at the same temperature), “^a, b, c^” different letters in the same series indicate significant difference at *p* < 0.05.

Sample	Swelling Ratio		
	25 °C	30 °C	35 °C
SAP12	29.03 ± 2.13 ^b^	22.08 ± 0.69 ^c^	21.24 ± 0.56 ^b,c^
SAP13	28.47 ± 3.02 ^b^	23.25 ± 1.53 ^b,c^	22.89 ± 1.34 ^b^
SAP14	29.04 ± 1.85 ^b^	24.22 ± 2.22 ^b^	21.33 ± 1.54 ^b,c^
SAPK1	26.23 ± 0.55 ^b,c^	22.46 ± 1.12 ^b,c^	21.75 ± 0.83 ^b,c^
SAPK2	23.76 ± 1.99 ^c^	21.32 ± 0.95 ^b,c^	20.07 ± 0.97 ^c,d^
SAPK3	22.54 ± 1.21 ^c^	20.94 ± 0.95 ^c^	18.87 ± 0.67 ^d^
SA	42.64 ± 2.65 ^a^	49.93 ± 1.38 ^a^	52.90 ± 2.02 ^a^

**Table 4:** Diffusion exponent, kinetic constant, and mechanism for the release of spores.

Temperature (℃)	Formulation	*n*	K	Mechanism
25	SAP12	0.36	0.15	Normal Fickian
	SAP13	0.35	0.15	Normal Fickian
	SAP14	0.39	0.13	Normal Fickian
	SAPK1	0.45	0.08	Normal Fickian
	SAPK2	0.49	0.06	Normal Fickian
	SAPK3	0.48	0.09	Normal Fickian
	SA	0.42	0.11	Normal Fickian
30	SAP12	0.09	0.55	Normal Fickian
	SAP13	0.09	0.53	Normal Fickian
	SAP14	0.08	0.56	Normal Fickian
	SAPK1	0.11	0.51	Normal Fickian
	SAPK2	0.10	0.49	Normal Fickian
	SAPK3	0.09	0.46	Normal Fickian
	SA	0.24	0.26	Normal Fickian
35	SAP12	0.06	0.67	Normal Fickian
	SAP13	0.07	0.64	Normal Fickian
	SAP14	0.06	0.67	Normal Fickian
	SAPK1	0.06	0.58	Normal Fickian
	SAPK2	0.05	0.62	Normal Fickian
	SAPK3	0.08	0.60	Normal Fickian
	SA	0.18	0.35	Normal Fickian
